# Morphologic Severity of Atypia Is Predictive of Lung Cancer Diagnosis

**DOI:** 10.3390/cancers15020397

**Published:** 2023-01-07

**Authors:** Lee Ann Santore, Samantha Novotny, Robert Tseng, Mit Patel, Denise Albano, Ankit Dhamija, Henry Tannous, Barbara Nemesure, Kenneth R. Shroyer, Thomas Bilfinger

**Affiliations:** 1 Renaissance School of Medicine, Stony Brook University, Stony Brook, NY 11794, USA; 2 Yale School of Medicine, Yale University, New Haven, CT 06520, USA; 3 Stony Brook Chest Clinic, Stony Brook University Hospital, Stony Brook, NY 11794, USA; 4 Department of Surgery, Stony Brook University, Stony Brook, NY 11794, USA; 5 Department of Family, Population and Preventive, Medicine, Stony Brook University, Stony Brook, NY 11794, USA; 6 Department of Pathology, Stony Brook University, Stony Brook, NY 11794, USA

**Keywords:** atypia, lung cancer, lung nodule, diagnosis, morphology, cytology

## Abstract

**Simple Summary:**

Lung cancer patients are treated by multidisciplinary care teams which rely upon the primary care provider to coordinate care and explain next steps to patients. In patients with nodule sampling that results in cytologic atypia, it is important that the primary care provider has a thorough understanding of the implications of this diagnosis so that they can advise the patient of appropriate follow up. This study investigated the correlation of atypia severity with diagnosis of lung cancer or benign respiratory process. We found that atypia severity, smoking pack years, and modified Herder score independently predicted cancer diagnosis. Patients with severe atypia may benefit from repeat sampling of lung nodules for cytologic confirmation within one month due to their high likelihood of malignancy, while those with less severe atypia may be followed clinically.

**Abstract:**

In cytologic analysis of lung nodules, specimens classified as atypia cannot be definitively diagnosed as benign or malignant. Atypia patients are typically subject to additional procedures to obtain repeat samples, thus delaying diagnosis. We evaluate morphologic categories predictive of lung cancer in atypia patients. This retrospective study stratified patients evaluated for primary lung nodules based on cytologic diagnoses. Atypia patients were further stratified based on the most severe verbiage used to describe the atypical cytology. Logistic regressions and receiver operator characteristic curves were performed. Of 129 patients with cytologic atypia, 62.8% later had cytologically or histologically confirmed lung cancer and 37.2% had benign respiratory processes. Atypia severity significantly predicted final diagnosis even while controlling for pack years and modified Herder score (*p* = 0.012). Pack years, atypia severity, and modified Herder score predicted final diagnosis independently and while adjusting for covariates (all *p* < 0.001). This model generated a significantly improved area under the curve compared to pack years, atypia severity, and modified Herder score (all *p* < 0.001) alone. Patients with severe atypia may benefit from repeat sampling for cytologic confirmation within one month due to high likelihood of malignancy, while those with milder atypia may be followed clinically.

## 1. Introduction

Lung cancer accounts for nearly one-quarter of all cancer deaths and is the second most common cancer type in both men and women [1]. Patients with suspicious lung nodules are often evaluated via cytologic analysis to confirm or rule out malignancy [2,3]. However, those with equivocal findings in which cytologic analysis is not definitive for benign versus malignant lesions may be classified as atypia. Guidelines are lacking for management of patients following a diagnosis of atypia. This paucity of recommendations for identifying patients that are most likely to benefit from further diagnostic workup, including repeat sampling procedures, results in a wide variety of non-standardized clinical management practices. Intensive workups for lung nodules are associated with increased rates of procedural complications, greater exposure to radiation, and higher cost [4]. As there are disadvantages seen with more rigorous lung nodule evaluations, it is important to identify patients at increased risk for malignancy who may benefit from an intensive workup.

Diagnosing lung cancer in the primary care setting is particularly challenging as symptoms are often absent or non-specific [5]. A survey of both primary care physicians and pulmonologists reported barriers to lung cancer screening include insufficient time and staffing [6]. Multidisciplinary care teams including surgeons, oncologists, radiologists, pulmonologists, pathologists, and primary care providers have been shown to yield superior results in patients with lung cancer [7,8,9]. The primary care provider, as the team member with the strongest relationship with the patient, often takes the lead in terms of coordinating care and explaining next steps. However, 68% of these physicians desire more information on follow-up recommendations for patients with lung nodules and 51% felt that decision aids would be helpful in handling lung cancer screening [6]. Patients with atypia may present to their primary care physicians for management. Thus, it is important for primary care physicians, in addition to pulmonologists, to feel comfortable risk-stratifying atypia patients and identifying those who require additional workup.

Several factors may be particularly concerning for poor outcomes in patients with lung nodules. More severe atypia has been identified as a predictor of decreased survival [10]. In a sample of high-risk patients with extensive smoking history and chronic obstructive pulmonary disease, moderate or worse atypia was associated with increased risk of lung cancer [11]. Our previous work revealed that 75% of patients with atypia later received a diagnosis of lung cancer, and of these, over 75% were diagnosed with malignancy within 6 months of atypia identification [12]. Additionally, the association between atypia and lung cancer incidence was found to be strongest when samples were collected within 5 months before the diagnosis of cancer, suggesting that atypia is often a late finding [13].

The purpose of this study was to identify morphologic predictors of lung cancer in pulmonary cytology specimens, that could be used to provide guidance on management after the initial diagnosis of atypia.

## 2. Materials and Methods

This study retrospectively analyzed the electronic medical records of patients evaluated by the Lung Cancer Evaluation Center at the Stony Brook Cancer Center, Stony Brook, NY, USA, between 1 January 2010 and 31 May 2020. The study was approved by the Institutional Review Board of Stony Brook University (IRB2019-00596). Only patients being evaluated for primary lung nodules were included in this investigation. Patients with non-primary lung nodules, such as distant metastases to the lung, or extra parenchymal thoracic tumors, such as mesotheliomas or lymphomas, were excluded from the study.

**Figure 1:**
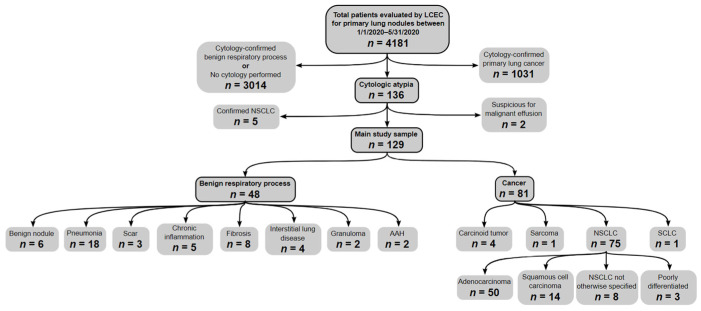
Study design flow chart. Note: LCEC = Lung cancer evaluation center. NSCLC = Non-small cell lung cancer. AAH = Atypical adenomatous hyperplasia. ILD = Interstitial lung disease. NOS = Not otherwise specified.

Patients were stratified based on initial cytologic diagnoses. Those with cytology reports confirming malignancy were classified as having confirmed primary lung cancers. Those with cytology reports that confirmed benign respiratory processes or whose presentations did not warrant the collection of a sample (i.e., high clinical suspicion for inflammatory lesion or pneumonia) were classified as having benign respiratory processes. Cytology reports that were not definitive for malignancy versus benign respiratory process and included one or more of the following phrases were classified as having atypia: atypia favoring reactive changes, rare atypia, mild atypia, atypia, moderate atypia, severe atypia, atypical adenomatous hyperplasia, or atypia, suspicious for malignancy. Thus, otherwise indeterminate results due to low diagnostic yield or sampling errors were not included in this study. Patients with atypia were then further stratified into those who were subsequently diagnosed with cytology- (repeat sampling) or histology- (surgical specimen) confirmed lung cancer and those who were not (see Figure 1 for study design flow chart).

The atypia categories were grouped into four general categories, including atypia favoring reactive changes, mild atypia, moderate atypia, or severe atypia, based on the most severe verbiage used to describe the atypical cytology. The mild group included patients whose cytology reports included the phrases rare atypia or mild atypia. The moderate atypia group included patients whose cytology report included the phrases atypia or moderate atypia. Finally, the severe atypia group included patients whose cytology reports included the phrases severe atypia, atypical adenomatous hyperplasia, or atypia, suspicious for malignancy.

In addition to morphologic characteristics, the clinical and radiologic features of our sample were described by calculating the modified Herder score [14]. The Herder score is a clinical prediction model based on the model created by Swensen et al. [15] which includes patient age, current or former smoking history, history of extra-thoracic cancer within five years, nodule diameter, nodule location in an upper lobe, spiculated nodule, and with the addition of Positron Emission Tomography (PET) fluorodeoxyglucose (FDG) avidity [14]. In this study, the modified Herder score was calculated as described by Herder et al. [14]; however, Herder et al. described FDG uptake qualitatively (not performed, absent, faint, moderate, intense), while we assigned these qualitative descriptors to quantitative maximum standard uptake values (SUV) such that they could be scored by non-radiologists (absent: SUV < 1, faint: 1 < SUV < 2.5, moderate: 2.5 < SUV < 4, intense: SUV > 4) [16,17].

Most patients in this study received a PET scan as recommended for patients with a nodule suspicious for lung cancer [18,19,20,21]. Protocol at the Lung Cancer Evaluation Center at the Stony Brook Cancer Center calls for PET imaging from the skull to the mid-thigh for highly suspicious nodules. Some patients did not undergo a PET scan (*n* = 29) due to physician or patient preference. The patients in this study received their initial biopsy and PET-FDG imaging within 2 weeks based on scheduling availability.

Additional data collected for this study included age, gender, smoking history, date of atypia diagnosis, date of cancer diagnosis or last date of examination, and specimen type. Specimen types included samples obtained via fine needle aspiration (FNA), bronchial brushing, other biopsies performed during bronchoscopy, bronchoalveolar lavage (BAL), biopsies obtained via interventional radiology procedures, and pleural fluid collections. The method of sampling was decided by the multidisciplinary care team and was based largely on radiologic features. Our institution favors FNA for specimen collection if possible. BAL is routinely performed at the conclusion of bronchoscopic evaluation and is only used for diagnosis if FNA, bronchial brushing, and other biopsies performed via bronchoscopy are not possible or are inconclusive. Interventional radiology procedures used to obtain cytology specimens also included core needle biopsies.

### Data Analytic Plan

Descriptive statistics were used to compare demographic characteristics between patients with atypia who went on to be diagnosed with cytologically or histologically proven lung cancer and those who did not. The median time between initial atypia diagnosis and either cancer diagnosis or the end of the surveillance period was calculated. Chi-square, Fisher’s exact, and Mann–Whitney *U* tests were used to compare differences between atypia severity, modified Herder score, pack year smoking history, specimen type, and final diagnoses. Logistic regression was performed to evaluate the relationship between atypia severity and final diagnoses while accounting for variations in modified Herder score and pack year smoking history. Receiver operator characteristic curves (ROC) were generated for the atypia severity, modified Herder score, and pack year smoking history individually and while adjusting for covariates predicting final diagnosis. SPSS version 26 (IBM Corporation, Armonk, NY, USA) was used to perform all analyses.

## 3. Results

Of 129 patients with cytologic atypia, 81 (62.8%) were subsequently found to have cytologically or histologically confirmed lung cancer and 48 (37.2%) had benign respiratory processes. There were no significant differences regarding gender or age between patients with atypia who went on to be diagnosed with cytologically or histologically proven lung cancer and those who did not (Table 1). There were differences regarding sampling method such that patients with atypia who went on to be diagnosed with lung cancer were more often sampled via FNA and less likely sampled via BAL than those who did not (Table 1). Atypia severity was significantly associated with final diagnoses with varying sensitivity and specificity for cancer across each subgroup (Table 2). The median (interquartile range) time between initial atypia and cancer diagnosis was 40 (14.5, 146.5) days, while the median length of the surveillance period for patients never diagnosed with cancer was 796.5 (218.5, 1220.3) days.

Final diagnosis of cancer or benign respiratory process was significantly associated with pack years smoking history, atypia severity, and modified Herder score (Table 1). Patients with atypia who went on to be diagnosed with lung cancer had significantly more pack years smoking history (47.3 ± 37.8) than those who did not (23.6 ± 23.8, *p* = 0.002). Atypia severity favoring reactive changes were significantly more likely to be followed by a diagnosis of a benign respiratory process (27.1%) than malignancy (2.5%, *p* < 0.001). Similarly, atypia severity of severe was significantly more likely to be followed by a diagnosis of a malignancy (29.6%) than a benign respiratory process (4.2%, *p* < 0.001). Modified Herder scores were significantly higher in patients diagnosed with cancer (0.690 ± 0.290) than those who were not (0.446 ± 0.343, *p* < 0.001).

**Figure 2:**
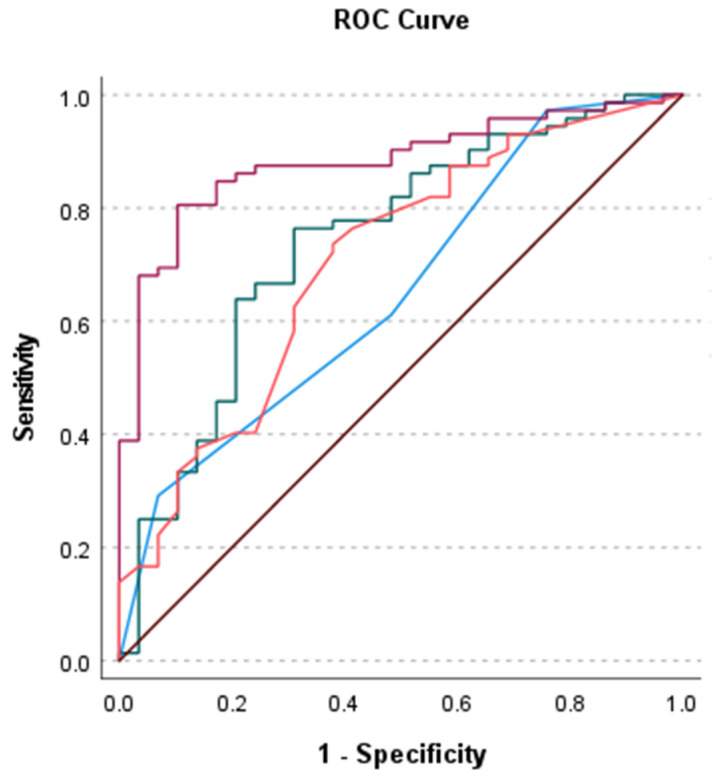
Receiver operator characteristic curves for atypia severity (blue), modified Herder score (green), pack years (orange) and model including atypia severity, modified Herder score, and pack years (magenta) predicting final diagnosis. Reference line (brown).

Atypia severity significantly predicted final diagnosis even while controlling for pack years and modified Herder score (*p* = 0.012). Pack years (c = 0.705 [95% CI 0.591, 0.819], *p* < 0.001), atypia severity (c = 0.653 [95% CI 0.541, 0.766], *p* < 0.001) and modified Herder score (c = 0.742 [95% CI 0.631, 0.853], *p* < 0.001) predicted final diagnosis independently and while adjusting for covariates (c = 0.881 [95% CI 0.813, 0.949], *p* < 0.001) with high discrimination (Table 3; Figure 2). There was no significant difference between the area under the curve produced by atypia severity and modified Herder score individually (*p* = 0.309), atypia severity and pack years individually (*p* = 0.510), or modified Herder score and pack years individually (*p* = 0.658). However, the model including pack years, atypia severity, and the modified Herder score generated a significantly improved area under the curve when compared to pack years (*p* = 0.002), atypia severity (*p* < 0.001) and the modified Herder score (*p* = 0.007) alone.

## 4. Discussion

This study reports several key findings. First, we highlighted atypia severity as a significant predictor of final diagnosis in patients with lung nodule sampling resulting in cytologic atypia. We additionally found atypia severity, pack-year smoking history, and modified Herder score were all associated with and were independent predictors of final diagnosis. Finally, we developed a model including atypia severity, pack-year smoking history, and modified Herder score that predicted malignancy with high discrimination while adjusting for covariates. This model performed better than models that used either pack year smoking history, modified Herder score, or atypia severity alone.

This suggests that severe atypia is useful in determining the next steps for clinical management of these patients, as those with severe atypia may benefit from repeat sampling, while those whose atypia favoring reactive changes may benefit from a more conservative course. The diagnosis of severe atypia highlights the need for clinical management from a multitude of care providers and repeat diagnostic procedures to establish a definitive diagnosis in these patients [10,11,12,13]. Notably, the relative probability of malignancy in patients with moderate or worse cytologic atypia was found to be high at the time of atypia diagnosis but decreased over time [22]. The association of severe atypia with malignancy was noted to be stronger for cases with recently diagnosed and persistent atypia [22]. Based on our findings that most patients with severe atypia are subsequently confirmed to have lung cancer, these patients may benefit from more aggressive clinical follow up.

Smoking history remains a key factor leading to a final diagnosis of malignancy. In recent years, the importance of smoking history in screening recommendations has increased. In 2021, official guidelines from the United States Preventive Services Task Force suggested lung cancer screening for all adults aged 55 to 80 who have a 20 pack-year smoking history [23]. While current guidelines suggest that screening should occur in those who have quit smoking in the past 15 years, there is evidence to suggest that the risk for lung cancer is still elevated in those who have quit in the past 25 years [24]. It is imperative that primary care physicians have frank conversations with their patients about smoking history. It has been shown that having conversations with patients rather than review of the electronic medical record can help identify more patients for lung cancer screening [25]. Physicians should keep such information in mind when assessing various evidence, such as sample atypia, for the possibility of malignancy.

The modified Herder score is a valuable prediction model used to help clinicians arrive at a diagnosis of a lung nodule. Originally created to incorporate FDG avidity on PET-CT, many groups have validated the accuracy of this model [16,26,27]. While the Herder score is a common tool used by radiologists, it may also be a useful metric for the entire care team to consider in accurately risk-stratifying and communicating with patients. One study found that patients with a high pretest probability of lung cancer and a high Herder score should be treated with the utmost urgency [28]. Understanding the Herder score may help the primary care physician and other members of the healthcare team escalate a patient’s care as needed and communicate with the patient in an appropriate and more informed manner.

Understanding the implications of atypia for lung cancer screening remains a challenge for primary care physicians and their patients. A patient’s decision to engage in lung cancer screening is heavily dependent on the attitudes, beliefs and values held by their physician towards screening tests [29,30]. With a strong relationship between atypia severity, smoking history, and modified Herder score to final diagnosis, the primary care team can feel more comfortable encouraging their patients to receive the screening recommendations. Importantly, since the model including atypia severity, modified Herder score, and pack years smoking history generated an area under the curve that was significantly improved compared to each individual factor, primary care providers should evaluate all three of these factors in concert when advising patients on the next steps in their management. Continued work on lung cancer screening procedures will help primary care physicians and patients make shared and informed decisions.

This study had several limitations. As an observational, retrospective study, data collection was limited as several variables were unavailable for analysis. Data records did not include molecular characteristics of biopsy samples and proceduralist and pathologist experience. There was also no inter-rater data between pathologists, whose verbiage for the initial atypia classification and final diagnoses may vary, although cytology and pathology reports are institutionally standardized. Small sample size and inclusion of patients at a single medical center may limit generalizability.

## 5. Conclusions

This study found that atypia severity, smoking history and modified Herder score were all independent factors significantly associated with a final diagnosis of cancer. Combining these three factors produced a better prediction model than using any individual factor. Future research is warranted to identify biomarkers for malignancy in patients deemed to have atypia on diagnostic evaluations as this, in concert with pack years smoking history, modified Herder score, and atypia morphology, may serve to improve diagnostic capabilities.

## Figures and Tables

**Table 1:** Sample characteristics and results of Chi-square, Fisher’s exact, and Mann–Whitney *U* tests.

		Total	Cancer	Benign
**Total** *n* (*%*)		129 (100%)	81 (62.8%)	48 (37.2%)
**Age** (*M + SD*)		66.53 ± 10.65	67.10 ± 9.16	65.56 ± 12.82
**Female** *n* (*%*)		63 (48.8%)	42 (51.9%)	21 (43.8%)
**Pack years** (*M + SD*)		40.51 ± 35.94	47.33 ± 37.83 **	23.59 ± 23.83 **
**Atypia severity** *n (%)*	*Favor reactive*	15 (11.6%)	2 (2.5%) ***	13 (27.1%) ***
	*Mild*	43 (33.3%)	29 (35.8%)	14 (29.2%)
	*Moderate*	45 (34.9%)	26 (32.1%)	19 (39.6%)
	*Severe*	26 (20.2%)	24 (29.6%) ***	2 (4.2%) ***
**Modified Herder score** (*M* ± *SD*)		0.600 ± 0.331	0.690 ± 0.290 ***	0.446 ± 0.343 ***
**Qualitative SUV***n (%*)	*Not performed*	29 (22.5%)	7 (8.6%) ***	22 (45.8%) ***
	*Absent (<1)*	0 (0%)	0 (0%)	0 (0%)
	*Faint (1–2.5)*	34 (26.4%)	21 (25.9%)	13 (27.1%)
	*Moderate (2.5–4)*	27 (20.9%)	17 (21.0%)	10 (20.8%)
	*Intense (>4)*	39 (30.2%)	36 (44.4%) ***	3 (6.3%) ***
**Maximum SUV** (*M* ± *SD*)		5.17 ± 5.27	6.01 ± 5.81 ***	2.78 ± 1.88 ***
**Sampling method** *n (%)*	*BAL*	16 (12.4%)	5 (6.2%) **	11 (22.9%) **
	*Brushing*	8 (6.2%)	5 (6.2%)	3 (6.3%)
	*IR*	9 (7.0%)	3 (3.7%)	6 (12.5%)
	*Bronch*	27 (20.9%)	16 (19.8%)	11 (22.9%)
	*FNA*	63 (48.8%)	47 (58.0%) **	16 (33.3%) **
	*Pleural fluid*	6 (4.7%)	5 (6.2%)	1 (2.1%)

Chi-square tests were performed for categorical variables with ≥5 samples in each cell. Fisher’s exact test was performed for categorical variables with <5 samples in each cell. Mann–Whitney *U* tests were performed for continuous variables. Cancer = patients with cytologically or histologically confirmed lung cancer. Benign = patients with cytologically or histologically confirmed benign lung process. M = mean. SD = standard deviation. BAL = Bronchial alveolar lavage. Brushing = Bronchial brushing. IR = sample obtained from interventional radiology procedure, including core needle biopsies. Bronch = tissue sample obtained through bronchoscopy procedure. FNA = fine needle aspiration. *** *p* < 0.001, ** *p* < 0.01.

**Table 2:** Sensitivity and specificity of atypia severity on the outcome of final diagnosis.

Final Diagnosis	Atypia Severity	Sensitivity	Specificity	False Positives	False Negatives
**Cancer**	Favor reactive changes	2.5%	72.9%	13	79
	Mild	35.8%	70.8%	14	52
	Moderate	32.1%	60.4%	19	55
	Severe	29.6%	**95.8%**	2	57
**Benign respiratory process**	Favor reactive changes	27.1%	**97.5%**	2	35
	Mild	29.2%	64.2%	29	34
	Moderate	39.6%	67.9%	26	29
	Severe	4.2%	70.4%	24	46

**Table 3:** Logistic regression predicting final diagnosis with area under the curve.

Variable	OR (95% CI)	c (95% CI)
Pack-year-smoking history	0.97 (0.95, 0.99) *	0.71 (0.59, 0.82) ***
Modified Herder score	0.05 (0.01, 0.28) ***	0.74 (0.63, 0.85) ***
Atypia severity	-	0.65 (0.54, 0.77) ***
*Mild*	46.66 (4.11, 530.45) **	-
*Moderate*	3.55 (0.56, 22.56)	-
*Severe*	8.80 (1.39, 55.66) *	-
Model including Pack-year-smoking history, Modified Herder score, and Atypia severity	-	0.88 (0.81, 0.95) ***

OR = odds ratio. CI = confidence interval. c = c-statistic, area under the curve. *** *p* < 0.001, ** *p* < 0.01, * *p* < 0.05.

## Data Availability

The data presented in this study are available on request from the corresponding author. The data are not publicly available due to patient privacy.
